# The effectiveness of *Hope Groups,* a mental health, parenting support, and violence prevention program for families affected by the war in Ukraine: Findings from a pre-post study

**DOI:** 10.1016/j.jmh.2024.100251

**Published:** 2024-07-23

**Authors:** Susan Hillis, Sydney Tucker, Nicole Baldonado, Evgenia Taradaika, Lyudmyla Bryn, Svitlana Kharchenko, Tetiana Machabelii, Roisin Taylor, Phil Green, Philip Goldman, Isang Awah, Joshua Baldonado, Praveen Gomez, Seth Flaxman, Oliver Ratmann, Jamie M. Lachman, Andres Villaveces, Lorraine Sherr, Lucie Cluver

**Affiliations:** a Global Reference Group on Children Affected by Crisis, University of Oxford, Oxford, United Kingdom; b Centre for Evidence-Based Social Intervention, Department of Social Policy and Intervention, University of Oxford, Oxford, United Kingdom; c World Without Orphans, Ukraine; d Children's Mission, Ukraine; e Ukraine Without Orphans, Ukraine; f Nehemiah, Ukraine; g VIVA International, India; h Maestral International, United States; i Department of Computer Science, University of Oxford, Oxford, United Kingdom; j Department of Mathematics, Imperial College London, United Kingdom; k Parenting for Lifelong Health, United Kingdom; l Centre of Social Science Research, University of Cape Town, Cape Town, South Africa; m Division of Violence Prevention, U.S. Centers for Disease Control and Prevention, Atlanta, GA, United States; n University College London, United Kingdom; o Department of Psychiatry, University of Cape Town, Cape Town, South Africa

**Keywords:** War, Armed conflict, Refugees, Internally displaced, Children, Abuse, Violence, Intervention, Psychosocial, Parenting, Mental health

## Abstract

**Background:**

Nearly one in six children lived in war zones in 2023. Evidence-based psychosocial and parenting support has potential to mitigate negative impacts for parents and children co-exposed to war and displacement, especially in relation to mental health and harsh parenting reactions. In the current war in Ukraine, local mental health experts co-created and evaluated, with global experts, the effectiveness of psychosocial and parenting support groups, called *‘Hope Groups’* on improvements in mental health, positive parenting, and violence against children. This paper aimed to assess the effectiveness of psychosocial and parenting support groups, called 'Hope Groups,' on improvements in caregiver mental health, positive parenting, and prevention of violence against children, for families affected by the war in Ukraine, using a pre/post study design.

**Methods:**

Participants (*n* = 577) included Ukrainian caregivers, 66% (381) of whom were parents and co-residing caregivers of children ages 0–17, while the remaining 34% were non-resident informal caregivers. Internally displaced, externally displaced, and those living at-home in war-torn regions were invited to groups by trained Ukrainian peer facilitators. Using a pre-post design, we compared individual level frequency measures at three time-points – baseline, midline, and endline, to assess changes in 4 mental health, and 9 parenting and child health outcomes. We analyzed these outcomes using paired *t*-tests to compare outcomes at baseline-to-midline (after 4-sessions) and baseline-to-endline (after 10-sessions), which estimated the mean changes in days per week and associated percent change, during the respective periods; we quantified uncertainties using bias-corrected and accelerated (BCa) bootstrapping with 95% uncertainty ranges for baseline-midline and baseline-endline estimates. We used this same approach for stratified analyses to assess potential effect modification by displacement status and facilitator type. We further used linear models to adjust for age and sex.

**Findings:**

Compared to baseline, every mental health, parenting, and child health outcome improved significantly at midline and endline. Mental health ratings showed endline reductions in depressive symptoms of 56.8% (95% CI: -59.0,-54.3; -1.8 days/week), and increases in hopefulness, coping with grief, and self-care, ranging from 62.0% (95% CI: 53.6,71.3; 2.2 days/week) to 77.0% (95% CI: 66.3,88.3; 2.2 days/week). Significant improvements in parenting and child health outcomes included monitoring children, reinforcing positive behavior, supporting child development, protecting child, nonviolent discipline, and child verbalizing emotions. By endline, emotional violence, physical violence, and child despondency had dropped by 57.7% (95% CI: -63.0%,-51.9; -1.3 days/week), 64.0% (95% CI: -79.0,-39.5; -0.22 days/week), and 51.9% (95% CI: -45.1,-57.9; -1.2 days/week), respectively. Outcomes stratified by displacement status remained significant across all groups, as did those according to facilitator type (lay versus professional).

**Interpretation:**

This study demonstrates preliminary evidence, using a brief survey and pre-post design as is appropriate for acute and early protracted emergency settings, of the feasibility and effectiveness of Hope Groups for war-affected Ukrainian caregivers, on improved mental health, positive parenting, and reduced violence against children.

## Background

Nearly one in six children live in war zones in 2023 (Østby et al., 2022), and over 2 billion people live in countries affected by conflict, fragility, or violence (Social Cohesion and Resilience 2024). In Ukraine alone, Russia's full-scale invasion since February 2022, forced over 13 million Ukrainians, mostly women and children, to flee their homes during the first 12 months of war (United Nations High Commissioner for Refugees [UNHCR] 2023a). The conflict has caused mass casualties, separation of families, loss of homes, and economic upheaval (United Nations High Commissioner for Refugees [UNHCR] 2023b; International Organization for Migration [IOM] 2022; Hinchey et al., 2023; Bogic et al., 2015). By June 2023, 6.3 million Ukrainians were externally displaced outside their country, and 5 million more remained internally displaced within Ukraine (United Nations High Commissioner for Refugees [UNHCR] 2023b). Missile, rocket, and drone attacks continue threatening lives of families and children, and destruction to infrastructure means loss of heat, gas, electricity, and water (International Organization for Migration [IOM] 2022; Hinchey et al., 2023). As the war continues, 4.5 million displaced Ukrainian women and children have returned to Ukraine to a highly uncertain future (International Organization for Migration [IOM] 2022; Bogic et al., 2015).

Exposure to war may lead to long-term consequences on health and well-being for children and families (UNHCR. UNICEF, UNDP, UNFPA 2022), such as mental health distress, complex grief, heightened exposure to violence, and exploitation (Boelen and Smid, 2017; Eltanamly et al., 2021; Morina et al., 2011; United Nations Children's Fund [UNICEF] 2022). The World Health Organization (WHO) estimates that 22.1% of any conflict-affected population experiences a mental disorder (United Nations High Commissioner for Refugees [UNHCR] 2023b). At the individual level, symptoms worsen over time, and children are among the most vulnerable (Hillis et al., 2017). Almost two of every three Ukrainian children were displaced, often separated from their fathers as they fled with their mothers (UNICEF 2022). These risks, considered adverse childhood experiences (ACEs), may have enduring consequences, including depression, suicide ideation, post-traumatic stress disorder, violence, school drop-out, insecure housing, and subsequent chronic and infectious diseases (Hillis et al., 2017). UNICEF demonstrated that in regions affected by the 2014 Russian invasion of Eastern Ukraine, psychosocial support programs could be effectively delivered to school children (Office of the Prosecutor General of Ukraine 2023). However, now an entire nation of Ukrainian children faces marked educational challenges, with vast damage to schools and ongoing threats causing 40% of students to be enrolled online (January 2023), (Bogdanov et al., 2017; Ukraine Children's Action Project 2023) These conditions place high demands on parents and teachers to provide educational support amidst blackouts, internet outages, and increased exposures to violence (Hillis et al., 2016a; Gradus 2022). Overall, 75% of Ukrainian parents state their children show symptoms of psychological traumatization (Calam et al., 2022).

Both theory and evidence suggest (Awuah et al., 2022) that strengthening mental health, parenting skills, and relationships between co-exposed parents and children in times of war is important for building resilience in children (Cluver et al., 2022). A systematic review of 38 studies among ‘co-exposed’ parents and children living together in war zones (displaced and non-displaced) found that war-time parenting practices showed less warmth (e.g., decreased praise or playing with children), and greater hostility (e.g., increased verbal or physical violence); these negative parenting practices were associated with increased child maladjustments, including post-traumatic stress symptoms, depression, and anxiety (Eltanamly et al., 2021). War-time threats and uncertainties present immense challenges in delivering flexible, contextualized, and scalable mental health, positive parenting. and violence prevention support programs to traumatized caregivers (Gonçalves Júnior et al., 2022). Although such programs can play a pivotal role in improving outcomes for caregivers and children amidst traumatizing circumstances, (Parenting for lifelong health 2024) little is known about feasible approaches for delivering such evidence-based programming in the Ukrainian context (Arega, 2023; Gardner et al., 2021).

To explore perceived need for and potential approaches to delivering such programming, several members of our investigation team (NB, SH, LB) participated in an invited consultation with displaced Ukrainian women living in refugee shelters bordering Ukraine, from April 16–30, 2022 – six weeks after the full-scale invasion. Our aims were to assess the urgent needs of the affected populations and to identify an intervention that could be co-created with the affected population to address their needs. Ukrainian women expressed an urgent need for community, parenting support, and mental health resources to equip them to care for their children and to “find hope again”. They recommended that many skilled highly educated mothers and caregivers (e.g. teachers, nurses, social workers, psychologists, architects, etc.) were unemployed, living in shelters, and could be trained to lead psychosocial support groups based on evidence-based content. They also identified strategies for strengthening acceptability of such groups, in the context of cultural unfamiliarity with this type of psychosocial programming.

We used a community-based participatory research process to design a three-phase *Hope Groups* program model to deliver evidence-based psychosocial and parenting support for families affected by acute or protracted wartime crisis. The *Hope Groups* program design team was co-led by social workers, researchers and psychologists from Ukraine, University of Oxford, World Without Orphans, and Imperial College London, in collaboration with Parenting for Lifelong Health (PLH) at the University of Oxford and VIVA. The design drew on evidence-based interventions with adaptations to the reality of the current group (Gardner et al., 2021; Covid-19: 24/7 parenting [Internet] 2023; VIVA Network: Guidance for Viva Partner networks [internet] 2023; Nguyen et al., 2023). Phases varied in delivery intensity: *Phase I -* Ukraine Parenting Tips sheets or social media messages, designed for the first 4 weeks of crisis onset, strengthen positive parenting (VIVA Network: Guidance for Viva Partner networks [internet] 2023) and reached over 9 million persons in Ukraine by April of 2022; (Nguyen et al., 2023) *Phase II – Hope Group* sessions 1–4, designed for acute and protracted crisis; and *Phase III – Hope Group* sessions 5–10, designed to help families living in protracted crisis transition to a new reality (Table 1). This study evaluated Phases II and III above, delivered as one continuous program called ‘*Hope Groups’*.

**Table 1:** Hope group study sessions and content descriptions.

Session	Title	Content
1	**Finding Stability - Our Everyday Tools**	This session builds skills on strategies for building hope in transition and crisis; we create discussion around the impacts of trauma and equip participants to begin building a personal “toolbox” for responding to crisis.
2	**Finding Stability - Our Anchoring Tools**	In this session, we teach common physical, emotional, behavioral, and cognitive reactions to trauma, and we build participant skills in developing stabilization techniques (such as deep breathing and progressive muscle relaxation) for coping with stress.
3	**Talk About It**	This session builds skills in identifying and sharing emotions participants are experiencing related to war-time events. We build skills in how to talk with children about war and loss.
4	**Strong Families**	This session builds skills on developing and maintaining strong relationships within families, particularly caregiver/child relationships. Skills are built for positive communication within relationships.
5	**Staying Safe Together**	This session builds skills and tools to be utilized to stay safe during crises and war, including together physically together as a family, when possible, as well as awareness of the risks and signs of human trafficking. We provide a guide for caregivers to learn how to talk with their children about human trafficking in age-appropriate terms, and we equip caregivers to safeguard their children's childhood even amidst crises.
6	**Staying Safe at Home**	This session builds skills to respond to strong emotions (such as anger) in healthy, safe ways – even in stressful moments. We discuss positive discipline of children.
7	**Coping with Loss**	This session teaches participants about the stages of grief, with an aim to normalize and remove stigma from the concept of grief. Participants build skills for healthy grieving, finding meaning in their new reality, and helping their children honor whom/what they lost.
8	**Building Hope through Understanding Guilt and Secondary Trauma**	This session equips participants to understand ‘survivor guilt’ and ‘secondary trauma’. Facilitators validate the stress of helping others in crisis and help participants build resilience amidst crises.
9	**Learning Together**	This session builds skills for caregivers to support their children's learning, amidst the war crisis, when many children have left their former schools and are attend new schools (even in a foreign language) or are studying at home/online without a traditional education environment.
10	**Resilience**	This final session builds skills in exploring emotions and discerning how to continually care for their own mental health, as well as their children's, as they build hope for the future amidst the war crisis.

The *Hope Groups* conceptual model (Supplementary Material, Diagram) shows that the intervention aims to strengthen validated outcomes measuring caregiver mental health, parenting skills, and child protection skills, all of which may be adversely affected by war exposures and displacement stressors. By strengthening caregiver mental health and parenting and child protection skills, the conceptual model shows that key aims are to reduce violence against children, and to improve both children's and caregivers’ wellbeing.

We used the TIDieR (Template for Intervention Description and Replication) Reporting Guidelines (Hoffman, no date). Hope Groups were designed as peer-led, semi-structured psychosocial and parenting support groups to build mental health and parenting skills over 10 sessions, taking into consideration lessons learned during the COVID-19 pandemic (Arega, 2023). Mental health content in the sessions used key principles for psychosocial support in armed conflict to build participant skills in developing new and strengthening existing coping approaches, stress reduction, and self-care, to address war-time challenges (El-Khani et al., 2022). Caregiving and violence prevention content of *Hope Groups* was based on global programs developed by Parenting for Lifelong Health (PLH) at the University of Oxford, as endorsed by WHO and UNICEF, with evidence of effectiveness from 15 randomized trials (El-Khani et al., 2021). Content was also informed by the ‘Ukraine Parenting’ resources (www.ukraineparenting.com) launched by the Global Initiative to Support Parents, WHO, PLH, UNICEF, United Nations High Commissioner for Refugees (UNHCR), United Nations Office on Drugs and Crime, Global Partnership to End Violence Against Children, and Early Childhood Development Action Network (UNICEF 2021; University of Oxford 2024). Displaced Ukrainian partners adapted all content to be appropriate for Ukrainian culture and decided key implementation strategies such as recruitment methods, size of support groups (3–5 people, respecting cultural preferences), inclusion of informal non-resident caregivers, and the intervention's name: “We should call the intervention ‘Hope Groups’”, they said, “because what we really need is a way to find hope again.” Session frequency and timing varied according to preferences of participants, occurring typically once or twice weekly for 90 min. Each participant received a printed ‘Hope Group Participant's Guide’ (WWO 2023).

In sum, we co-created and evaluated *Hope Groups*: a mental health, psychosocial, and parenting support group based on evidence-based content to strengthen Ukrainian parents and caregivers affected by war. We hypothesized that participation in this 10-session *Hope Groups* intervention among Ukrainian participants, who were internally displaced, living at-home in war-torn regions, or externally displaced across 8 countries would be associated with improvements in one or more outcomes for each of these categories of indicators: caregiver mental health, positive parenting, prevention of violence against children, and reported child wellbeing (Gardner et al., 2021). Mental health content utilizes key principles for psychosocial support in armed conflict to build participants’ skills in healthy grieving and coping, stress reduction, and self-care to address war-time challenges. Positive parenting content includes reinforcing positive behavior, supporting child development, child supervision and protection, and nonviolent discipline. Violence content includes skills for preventing physical and emotional violence against children (Table 1). If initial results are positive, *Hope Groups* could be further tested in randomized trials, and if effective, subsequently adapted by government, donors, Non-Governmental Organizations (NGOs), Faith-Based Organizations (FBOs), and faith and community networks in conflict contexts within and beyond Ukraine.

## Methods

### Study design, population and participants, settings

We used a pre/post design to obtain preliminary evidence on the feasibility and effectiveness of Hope Groups. Given the war setting, a pre/post pilot design for an initial evaluation was more feasible than a randomized design, while also providing data to guide future evaluations with a comparison arm. Thus, the primary independent variable was the 10-session Hope Group intervention, and the primary outcomes included four adult mental health outcomes, and nine parenting and child health outcomes. Measured potential confounders included age and sex, while potential effect modifiers included displacement status and facilitator type.

Trained peer facilitators recruited 701 participants from the study population of externally displaced, internally displaced, and home-dwelling Ukrainians affected by the Russian invasion, from November 2022 - June 2023, Our analytic sample included the 577 participants who provided follow-up data at midline or endline. Facilitators recruited through their organizations and networks, including NGOs, FBOs, refugee shelters, churches, and aid distribution sites, by engaging staff, influencers, leaders, and outreach workers within those networks. Eligibility criteria for participants included: 1. Ukrainian or another nationality affected by the war; 2. Understands the Ukrainian language’ 3. Co-residing parent or caregiver of children ages 0–17, or a non-resident caregiver who provides informal care to children; 4. Is 18 or older. Eligibility criteria for facilitators included: (1) is Ukrainian, or another nationality and affected by the war in Ukraine; (2) speaks and understands Ukrainian; (3) is 18 or older; (4) is connected to an NGO, refugee shelter, FBO, church, or other network of Ukrainians; (5) has had previous experience leading small group discussions; (6) self-defines as emotionally stable with capacity to support others; and (7) is willing to participate in *Hope Groups* trainings.

Our study was implemented in the setting of camps where displaced persons had recently found safety, as well as in acute war emergency settings with ongoing bombings, sirens, and shelling. Many of the displaced participants lived in shelters supported by UNHCR and FBOs in Poland and Western Ukraine. The locations for conducting the in-person Hope Groups sessions included NGOs, FBOs, shelters, churches, aid distribution sites, as well as homes or parks.

### Intervention development, contextualization, and organizational structure

We used the ADAPT guidance to develop and contextualize the Hope Groups (Moore et al., 2021). First, we created an adaptation team with diverse stakeholders, including experts (a Ukrainian psychologist specializing in child trauma and program development living in a war-torn city; a global senior technical advisor with two decades of research experience in Ukraine and Eastern Europe; and a social worker with young children who was displaced from Ukraine and had expertise in community programming); the leadership of several hotel-shelters near Krakow housing nearly 1000 Ukrainian mothers and children; and five displaced Ukrainian mothers.

During the initial phase, the adaptation team conducted approximately 12 consultation sessions with 40 displaced Ukrainian residents, in Ukrainian or Russian, to assess their key needs. Topics for the 10 *Hope Groups* sessions (Table 1) were based on these expressed needs. The adaptation team determined that evidence-based mental health and parenting resources were available to be adapted for the war in Ukraine, but a delivery mechanism needed to be developed for cultural relevance in the context of war and displacement.

To create adaptations, the adaptation team developed each of the 10 sessions for the interactive *Hope Groups* Facilitator's Guide, each of which was reviewed and revised to ensure alignment with the evidence. A small advisory group of displaced Ukrainians interested in becoming ‘*Hope Groups’* facilitators helped identify potential constraints; adjust language to ensure culturally sensitive terminology; ensure contextually appealing design; and address war-time barriers to participation, to ensure usage of a feasible delivery mechanism for *Hope Groups*. This advisory group suggested increasing uptake by including flexible delivery options; ensuring a realistic number and duration of sessions; and using word-of-mouth recruitment within local partners and networks, to overcome cultural bias against mental health care and support groups. Each session was piloted with the advisory group, and further adapted to optimize relevance. In sum, each aspect of the intervention and delivery development was guided and informed by displaced persons from Ukraine, based on evidence-based content.

The *Hope Groups* organizational structure included a program director, team of coordinators, and group facilitators. The program director served as the Master Trainer and used a standardized guide to provide 8 h of training for 5 coordinators and 39 Ukrainian facilitators either virtually or in-person. The program director used regular online meetings, telephone and/or text message communication with the team of coordinators to discuss session-specific successes and challenges. Each coordinator supervised a small group of facilitators using these same communication platforms. To strengthen implementation fidelity, all *Hope Groups* staff underwent systematic training on program content for each of the 10 sessions, most sessions were delivered with reported fidelity, in terms of content and frequency. Sessions that were delayed by illness, intense bombing, or re-entry to Ukraine were typically rescheduled within 1- 2 weeks or held online.

### Procedures

We collected baseline, midline, and endline data via self-administered surveys on personal smartphones, adapting questions from validated scales (Table S1). Crisis adaptations essential for the war context ensured the survey was short, non-triggering, and culturally appropriate. The University of Oxford Ethics Committee approved this study (Approval #R84832/RE001). Informed consent was obtained on web-based links using mobile phones. As flexibility and convenience were vital to successful implementation and evaluation in an ever-changing war and displacement situation, all outcomes were also self-reported using standardized measures on mobile phones. *Hope Groups* delivery occurred in-person, virtually, or in hybrid format, led by mental health professionals or lay-trained facilitators . No incentives were offered, and confidentiality was maintained. Our protocol required immediate referrals to child protection and health services in the event any participant reported severe abuse, rape, or other significant harm. However, no such referrals were needed. Ukrainian mental health experts translated all materials into Ukrainian.

### Outcomes

Each outcome used continuous frequency rating scales, with single item responses, to assess mean change and percent change in ‘days in the last week.’ This approach was used to strengthen recall amidst ongoing distress. For all participants, we measured four outcome ratings related to adult mental health: depression score, coping with grief, hopefulness about the future, and self-care practices (Supplementary Table 1). For parents and caregivers co-residing with children, we further measured nine parent and child health outcomes across three main categories: (a) positive parenting (5 outcomes included monitoring child, reinforcing positive behavior, supporting child development, child protection, nonviolent discipline); (b) violence against children (2 outcomes included physical violence and emotional violence); and (c) child wellbeing (2 outcomes included reported child despondency and child verbalizing emotions) (Supplementary Table 1). For the two outcomes focused on child wellbeing, parents were asked to report on their oldest child. For the positive parenting measures, parents reported on improved outcomes for one or more of their children.

Our use of a brief, rather than in-depth survey to measure outcomes of interest was influenced by the United Nations High Commission on Refugees (UNHCR) and United Nations Children's Fund (UNICEF) policy brief on challenges and solutions for conducting research in emergency and humanitarian settings (UNHCR 2021). In using a brief survey, we also aimed to limit threats to data validity that may be linked in war-affected settings, to high stress, elevated trauma, mental fatigue, difficulty concentrating, and reduced ability to focus (Ben-Ezra et al., 2023). To further strengthen validity of our survey measures in the Ukrainian context, a native Ukrainian speaker who is bilingual translated the questions from English into Ukrainian. Next, to ensure understanding and cultural fit of the questions in the Ukrainian war context, the program director, along with the 5 Ukrainian study coordinators, reviewed the survey measures in Ukrainian for logic and fit, and suggested edits for clarity, culture, and contextual fit.

As shown in Table S1, positive parenting measures were based on parent report of the widely used and well-validated Alabama Parenting Questionnaire with previous use in several studies in South Africa, as well as several other validated measures (Table S1) (Kyriazos and Stalikas, 2019; Cluver et al., 2017; Lachman et al., 2014). The two violence against children measures were based on the International Society for Prevention of Child Abuse and Neglect (IPSCAN) parent version of the International Child Abuse Screening Tool (ICAST-P). This Tool has been field tested in eight countries, reviewed internationally using the Delphi process (Meinck et al., 2021).

We powered our study based on violence against children. Based on the estimated effect size for evidence-based positive parenting on this outcome, we estimated that 235 parent and co-residing caregiver participants would be sufficient to detect significant effects with 80% power, under the assumption of a 20% loss to follow-up. We estimated prevalence of any type of violence as 40% before exposure to the intervention, and we estimated that the intervention would reduce this outcome by half (Hillis et al., 2016a; WHO 2021). Given our interest in possible effect modification of violence against children by displacement status or delivery type (in-person versus virtual), we aimed to enroll 475 parents and co-residing caregivers. For our full sample that additionally included informal non-resident caregivers, we aimed to enroll 700 participants to detect significant effects in one or more mental health outcomes, with 80% power (United Nations High Commissioner for Refugees [UNHCR] 2023b).

### Data cleaning

All data was cleaned and analyzed in R (version 2023.03.0). For those who participated beyond initial enrollment and provided follow-up data at midline and/or endline, missing data on a given outcome were excluded from the analyses of that outcome. Overall, numbers of the study population with missing outcome data were low, as shown by our outcome-specific totals (Shown in Table 3). As our survey was designed to collect data on occurrence of outcomes in the past week, all outcome variables ranged from 0 to 7, and scaling was not needed.

### Analytic approach

#### Primary analyses

We first independently analyzed individual level outcomes using paired *t*-tests (R package Stats, v 4.3.1) to compare participant outcomes at baseline-to-midline (after 4-sessions) and baseline-to-endline (after 10-sessions), which estimated the mean changes in days per week and percent change associated with participation in Hope Groups. Statistical uncertainty was quantified using bias-corrected and accelerated (BCa) bootstrapping with 95% bootstrap uncertainty ranges for baseline-midline and baseline-endline estimates to account for non-normal distributions (R package “Boot”, v 1.3–28.1). Secondly, in order to adjust for potential confounders, we then constructed linear models to assess changes in outcomes associated with Hope Group participation, adjusted for age and sex, and including a random effect on both facilitator and participant to account for clustering. As linear regression and paired *t*-tests are expected to yield consistent results, any differences in results would reveal the presence of confounding.

#### Stratified analyses

We also employed paired *t*-tests stratified by displacement status (internal, external and non-displaced), to examine potential differences in intervention effect between sub-groups. To assess potential effect modification by facilitator type, we used linear models regressing all outcomes on the interaction between Hope Group participation and facilitator training (mental health professional versus lay leader), with a random effect on each participant and coding time as a categorical variable indicating baseline, midline, or endline. Models were not adjusted for covariates, in order to yield both the estimated change in outcomes within the referent group (lay-trained) and the additional change within the groups led by professionally trained facilitators.

#### Sensitivity analyses

As a sensitivity analysis to our assumption that outcome data are normally distributed, we considered the number of days the outcome occurred in the past week as a count and constructed analogous Poisson models using the lme4 package in R to assess if results remained consistent. We estimated incidence rate ratios (IRRs) by regressing all thirteen outcomes on participation in Hope Groups, with a random effect on each participant to account for clustering. Over dispersion was assessed prior to proceeding with Poisson models. We compared the magnitude and direction of IRRs with our primary results from paired *t*-tests.

Given the absence of a control group in this pre-post study, we investigated if temporal trends occurred over the study duration that may have influenced pre-post results linked to the intervention. To investigate if participant outcomes improved over time independently of the intervention, we used *t*-tests to compare baseline outcome measures during the first half of the recruitment period (November 2022-March 2023) to the second half of the recruitment period (March 2023-July 2023) and assess whether statistically significant differences in baseline outcomes were observed over the duration of our study.

### Role of the funding source

The funders of the study had no role in study design, data collection, data analysis, data interpretation, or writing of the report.

## Results

Baseline surveys were completed by 701 participants, and 577 participants (82%) completed baseline, midline, and/or endline surveys. Therefore, our final analytical sample included 577 Ukrainian caregivers affected by war, 66% (381) of whom were parents and co-residing caregivers, while the remaining 34% were non-resident informal caregivers (Table 2). Participants were aged 20–29 (29.1%), 40–49 (26.7%), or 60+ (21.7%), and 94.2% were female. Participants were internally displaced within Ukraine (50.8%), externally displaced outside of Ukraine (27.6%), or living at-home in war-torn areas (21.5%). Most participants were in Ukraine (69%) or Poland (25%), while the remaining 6% were in 6 other countries throughout Europe. An average of 2.4 children (ages 0–17) per parent were reported. Although data on children's age were not available, facilitators who recruited from the largest refugee shelters reported observing the full range of children, widely distributed across age groups from infants/toddlers, preschoolers, elementary age, and preadolescent and adolescents. In-person Hope Groups were preferred (78%) over virtual or hybrid delivery (21.1%); 63.3% of participants had a facilitator with prior training in mental health, counseling, or social work, and the remainder had facilitators drawn from lay populations also affected by the war.

**Table 2:** Characteristics of Ukrainian “Hope Group” Participants from 2022 to 2023.

	Parents and Co-residing Caregiver (*N* = 381)	Non-resident Informal Caregivers (*N* = 196)	Total Sample (*N* = 577)
**Age**			
18–19	0 (0%)	12 (6.1%)	12 (2.1%)
20–29	23 (6.0%)	25 (12.8%)	48 (8.3%)
30–39	148 (38.8%)	20 (10.2%)	168 (29.1%)
40–49	132 (34.6%)	22 (11.2%)	154 (26.7%)
50–59	37 (9.7%)	32 (16.3%)	69 (12.0%)
60+	40 (10.5%)	85 (43.4%)	125 (21.7%)
**Sex**			
Female	359 (94.2%)	165 (84.2%)	524 (90.8%)
Male	21 (5.5%)	31 (15.8%)	52 (9.0%)
**Displacement Status**			
Externally Displaced (in a country other than Ukraine)	116 (30.4%)	43 (21.9%)	159 (27.6%)
Internally Displaced (in Ukraine)	179 (47.0%)	114 (58.2%)	293 (50.8%)
Living at Home (not displaced)	85 (22.3%)	39 (19.9%)	124 (21.5%)
**Country Location**			
Cyprus	10 (2.6%)	6 (3.1%)	16 (2.8%)
Germany	6 (1.6%)	1 (0.5%)	7 (1.2%)
Italy	1 (0.3%)	0 (0%)	1 (0.2%)
Poland	103 (27.0%)	41 (20.9%)	144 (25.0%)
Ukraine	256 (67.2%)	142 (72.4%)	398 (69.0%)
United Kingdom	5 (1.3%)	1 (0.5%)	6 (1.0%)
Belgium	0 (0%)	1 (0.5%)	1 (0.2%)
Spain	0 (0%)	3 (1.5%)	3 (0.5%)
**Session Format**			
In-person	280 (73.5%)	170 (86.7%)	450 (78.0%)
Remote meetings	98 (25.7%)	24 (12.2%)	122 (21.1%)

**Table 3:** Primary Paired T-Test Results Among Ukrainian "Hope Group" Participants in 2022–2023, Comparing Baseline-Midline and Baseline-Endline.

	Baseline to Midline				Baseline to Endline			
	N	Mean Difference in Days (95% CI)	P-value	% Change (95% CI)	N	Mean Difference in Days (95% CI)	P-value	% Change (95% CI)
**Adult Mental Health** **(Full Sample, *N*=577)**								
Depression rating	576^	−1.25 (−1.42, −1.08)	<0.001***	−38.25% (−42.24, −34.04)	554^	−1.84 (−2.04, −1.64)	<0.001***	−56.78% (−59.01, −54.32)
Self-Care Practices	575^	1.29 (1.11, 1.46)	<0.001***	44.86% (37.16, 53.23)	551^	2.21 (2.00, 2.41)	<0.001***	76.96% (66.25, 88.26)
Hopefulness about Future	577^	1.15 (0.97, 1.34)	<0.001***	33.96% (27.46, 41.22)	552^	2.21 (1.99, 2.41)	<0.001***	61.96% (53.62, 71.28)
Coping with Grief	571^	0.91 (0.69, 1.11)	<0.001***	37.72% (27.07, 48.52)	546^	1.56 (1.32, 1.80)	<0.001***	66.51% (53.45, 80.37)
**Parent and Child Health** **(Parents and Co-residing Caregivers Only, *N*=381)**								
Physical Violence	365^	−0.12 (−0.23, −0.011)	.039*	−33.08% (−53.9, −1.13)	355^	−0.22 (−0.33, −0.12)	<0.001***	−64.00% (−78.98, −38.53)
Emotional Violence	367^	−0.76 (−0.94, −0.58)	<0.001***	−33.90% (−39.96, −27.25)	357^	−1.32 (−1.52, −1.13)	<0.001***	−57.69% (−62.97, −51.90)
Nonviolent Discipline	366^	.59 (0.34, 0.82)	<0.001***	14.84% (8.37, ^21.48)	356^	1.09 (0.78, 1.37)	<0.001***	26.97% (18.74, 35.53)
Protecting Child	366^	.65 (0.43, 0.87)	<0.001***	13.28% (8.61, 18.44)	357^	1.40 (1.15, 1.64)	<0.001***	28.54% (22.41, 34.46)
Monitoring Child	367^	.87 (0.67, 1.09)	<0.001***	19.35% (14.33, 25.36)	356^	1.83 (1.58, 2.09)	<0.001***	39.01% (32.00, 47.15)
Supporting Child Development	368^	.66 (0.46, 0.85)	<0.001***	15.99% (10.78, 21.53)	358^	1.66 (1.43, 1.90)	<0.001***	39.54% (32.72, 47.49)
Reinforcing Positive Behavior	365^	.74 (0.56, 0.92)	<0.001***	17.15% (12.54, 22.24)	356*	1.62 (1.39, 1.85)	<0.001***	37.45% (30.90, 44.74)
Child Despondency	364^	−0.78 (−0.97, −0.59)	<0.001***	−33.37% (−39.40, −26.80)	357^	−1.19 (−1.41, −1.00)	<0.001***	−51.89% (−57.89, −45.09)
Child Verbalizing Emotions	363^	1.00 (0.77, 1.23)	<0.001***	29.16% (21.24, 37.85)	353^	1.57 (1.28, 1.87)	<0.001***	45.66% (34.47, 57.20)

⁎ Indicates level of statistical significance. Bias-corrected and accelerated(bCA) bootstrapping performed well with paired *t*-tests and poorly with linear regression; therefore, we use paired *t*-tests with bCA bootstrapping for unadjusted results and linear regression with percentile bootstrapping for adjusted results in Table S4; results remained consistent;.

^ Indicates sample size decreased due to missing data.

**Fig. 1:**
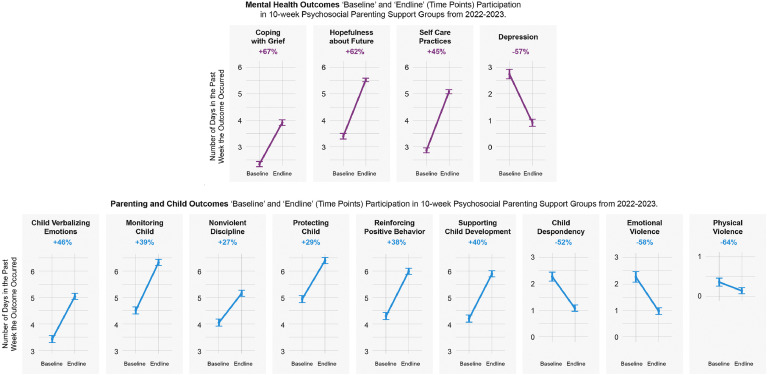
Ukrainian hope group baseline-endline results.

Our examination of the full 10-week intervention found statistically significant improvements across all mental health, parenting, and child outcomes when examining change from baseline-to-endline (Fig. 1). By endline, the frequency of self-care practices and hopefulness, as well as positive parenting behaviors, including reinforcing positive behaviors, monitoring child, nonviolent discipline, and protecting child had increased to 5 days per week or greater (Fig. 1). We also found significant improvement in all outcomes from baseline-to-midline, which increased and remained significant for baseline-to-endline measures (Table 3). For mental health outcomes, we found midline and endline reductions in depression ratings of 38.3% (95% CI −42.2, –34.0; −1.3 days/week) and 56.8% (95% CI −59.0, –54.3%; −1.8 days/week), respectively; and increases in hopefulness, coping with grief, and self-care ranging from 34.0% (95% CI 27.5, 41.2; 1.2 days//week) to 44.9% (95% CI 37.2, 53.2; 1.3 days/week) at midline; and 62.0% (95% CI 53.6%, 71.3%; 2.2 days//week) to 77.0% (95% CI 66.3%, 88.3%; 2.2 days/week) at endline.

All positive parenting outcomes also significantly improved among the 381parents and co-residing caregivers (Table 3), with respective increases from baseline-to-midline and baseline-to-endline of monitoring child by 19% (95% CI 14.3, 25.4; 0.87 days/week) and 39% (95% CI 32.0, 47.2; 1.8 days/week); protecting child by 13% (95% CI 8.6, 18.4; 0.65 days/week) and 29% (95% CI 22.4, 34.5; 1.4 days/week); nonviolent discipline by 15% (95% CI 8.4, 21.5; 0.59 days/week) and 27% (95% CI 18.7, 35.5; 1.1 days/week); and reinforcing positive behavior by 17% (95% CI 12.5, 22.2; 0.74 days/week) and 37% (95% CI 30.9, 34.7; 1.6 days/week. Occurrences of reported child outcomes also showed improvements with respective midline and endline decreases in emotional violence by 34% (95% CI −40.0, −27.3; −0.76 days/week) and 58% (95% CI −53.9, −63.0, - 1.3 days/week), physical violence by 33% (95% CI −59.9, −1.1; −0.12 days/week) and 64% (95% CI −38.5, −79.0; −0.23 days/week), and child despondency by 33% (95% CI −39.4, −26.8; −0.78 days/week) and 64% (95% CI 38.5, 79.0; −0.23 days/week). Child(ren)’s verbalizing emotions by expressing their problems and worries in meaningful conversations with their parent increased by 29% (95% CI 21.2, 37.9; 1.0 days/week) and 45.7% (95% CI 34.5, 57.2; 1.6 days/week), respectively. Results were unchanged when adjusting for age and sex and including a random effect on facilitator to account for facilitator-level clustering (Table S2).

**Fig. 2:**
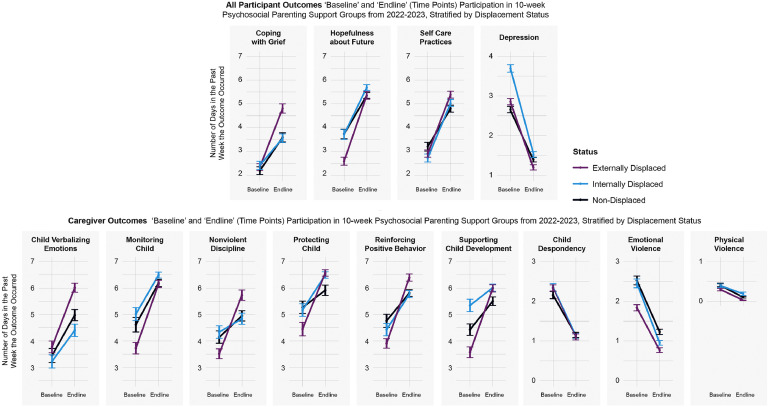
Ukrainian hope group baseline-endline results stratified by displacement status.

Findings stratified by displacement type (internally, externally, non-displaced) revealed that all mental health and parenting outcomes remained statistically significant for each displacement types from baseline-endline (Table 4), though larger improvements were found among displaced populations. On average, externally displaced participants had worse outcome levels at baseline, compared with internally displaced and non-displaced participants, and experienced greater relative improvements by endline for 10 outcomes (Fig. 2).

**Table 4:** Stratified Paired T-Test Results Among Ukrainian "Hope Group" Participants in 2022–2023, Comparing Baseline-Endline and Stratified by Displacement Status.

	Non-Displaced (At Home in Ukraine)		Internally Displaced (in Ukraine)		Externally Displaced (Outside Ukraine)	
	Estimate *(Absolute Change in Days per Week each Outcome Occurred)*	95% CI	Estimate *(Absolute Change in Days per Week each Outcome Occurred)*	95% CI	Estimate *(Absolute Change in Days per Week each Outcome Occurred)*	95% CI
**Adult Mental Health (Full Sample, *N*=577)**						
Depression rating	−1.26	(−1.71, −0.86)	−2.17	(−2.45, −1.89)	−1.66	(−2.00, −1.29)
Self-Care Practices	1.59	(1.17, 2.05)	2.31	(2.04, 2.60)	2.46	(2.06, 2.85)
Hopefulness about Future	1.61	(1.11, 2.02)	1.96	(1.67, 2.22)	2.77	(2.34, 3.16)
Coping with Grief	1.39	(0.88, 1.90)	1.14	(0.78, 1.45)	2.47	(2.00, 2.88)
**Parent and Child Health (Parents and Co-residing Caregivers Only, *N*=381)**						
Physical Violence	−0.30	(−0.65, −0.08)	−0.20	(−0.35, −0.01)	−0.27	(−0.44, −0.16)
Emotional Violence	−1.29	(−1.74, −0.84)	−1.49	(−1.81, −1.18)	−1.07	(−1.35, −0.85)
Monitoring Child	1.57	(1.05, 2.15)	1.48	(1.16, 1.81)	2.42	(2.00, 2.87)
Protecting Child	0.64	(0.14, 1.13)	1.32	(0.99, 1.65)	2.12	(1.72, 2.58)
Supporting Child Development	1.08	(0.63, 1.64)	1.47	(1.15, 1.82)	2.42	(2.03, 2.83)
Reinforcing Positive Behavior	1.04	(0.60, 1.62)	1.34	(1.03, 1.67)	2.49	(1.99, 2.85)
Nonviolent Discipline	0.81	(0.23, 1.36)	0.50	(0.05, 0.93)	2.21	(1.78, 2.68)
Child Despondency	−1.01	(−1.51, −0.58)	−1.22	(−1.53, −0.91)	−1.23	(−1.59, −0.87)
Child Verbalizing Emotions	1.53	(0.96, 2.19)	1.18	(0.73, 1.60)	2.24	(1.73, 2.73)

Linear models assessing interaction by facilitator background (mental health professional versus lay-facilitator) revealed that participants in both groups experienced significant improvements from baseline-endline across all mental health, parenting, and child outcomes, indicating Hope Groups are effective with lay-trained or professional trained facilitators. Of note, participants with mental health professional facilitators experienced additional improvements among all mental health outcomes and all parenting outcomes except physical violence, using a statistically significant interaction threshold of *p* < .05 (Table S3). Improvements in child outcomes (child despondency and child verbalizing emotions) did not significantly differ between lay-trained and professionally trained facilitators.

Results from Poisson models estimating incidence rate ratios (IRRs) at midline and endline remained consistent with paired *t*-test results (Table S4).

We found no statistically significant changes in baseline outcome measures during the second half of our eight-month study period, compared to the first half, suggesting that it was unlikely that temporal trends could have explained observed improvements in participant outcomes from baseline-midline and baseline-endline over the study duration (Table S5).

## Discussion

Delivery of the *Hope Group* intervention for Ukrainians during 2022–2023 of the Russian invasion led to significant improvements in mental health, positive parenting, and violence prevention outcomes. Strengthened mental health was shown by the 60% reduction in the frequency of participants’ depressive symptoms, over a 50% increase in hopefulness and coping with grief, and nearly an 80% improvement in self-care. Among parents and caregivers, over 50% reductions occurred in the frequency of physical abuse and 60%, in emotional abuse. Significant improvements occurred for all remaining outcomes reported by caregivers, including monitoring children, reinforcing positive behavior, supporting child development, child protection, nonviolent discipline; decreased child despondency and increased frequency of children verbalizing their emotions. After ten sessions, a two-fold improvement in all outcomes occurred, compared to midline. These findings suggest that *Hope Groups* were effective both as a 4-session *acute crisis transition* intervention and became more effective in strengthening mental health and positive parenting after the full 10-week *prolonged crisis transition* intervention.

WHO prevalence estimates show that over one in five people in post-conflict settings have mental health disorders (e.g., anxiety, depression, post-traumatic stress disorder, etc.) (United Nations Office on Drugs and Crime [UNODC] Ukraine 2022). Given the very high prevalence of these disorders, experts confirm the urgency in conflict settings of both strengthening sustainable mental health care, and implementing psychosocial interventions for war-affected populations that strengthen community care, self-help, and support (United Nations Office on Drugs and Crime [UNODC] Ukraine 2022). *Hope Groups* are an example of this latter type of intervention. Evidence further shows that mental health interventions in crisis contexts require cultural adaptation; can be effectively led by well-trained lay staff; and hold promise for expanded reach through virtual delivery (Charlson et al., 2019). *Hope Groups* development, implementation, and findings were aligned with this evidence (Soklaridis et al., 2020).

Our findings among war-affected parents caring for children across the developmental spectrum from birth to 17 years of age, showed that *Hope Groups* led to marked improvements in all outcomes for those internally displaced, externally displaced, and non-displaced. These benefits were observed among Hope Group participants led by both mental health professionals and lay-trained facilitators.

By comparison, a review of 14 experimental or quasi-experimental studies of parenting interventions among forcibly displaced populations (among Somali, South Sudanese, Burmese, Palestinian, Lebanese, and Bosnian groups) found evidence of effectiveness for improving caregiver mental health, positive parenting, psychosocial wellbeing, and/or parental stress management (Gillespie et al., 2022). For example, findings were promising across all these outcomes for the Caregiver Support Intervention pilot study implemented among Syrian refugees living in Lebanon (Miller et al., 2020). Beyond this, other programs showed mild to moderate improvement in some domains for parenting practices, child psychosocial outcomes, and parent mental health (Masten, 2021). To our knowledge, our *Hope Group* study is the first to evaluate a mental health, psychosocial support, and violence prevention intervention among displaced and non-displaced Ukrainians affected by the war. We found that *Hope Groups* improvements were more comprehensive and generally of greater magnitude than those reported in previous studies. We also note several valuable learning points of our work, namely the use of self-administered, shorter questionaries for mental health outcomes in conflict zones was feasible and appears informative; smartphones can provide an inexpensive and available platform for self-administered surveys; and hybrid and virtual platforms can be effectively used to deliver effective interventions in conflict zones.

We considered several limitations. First, because war circumstances presented a mental health and parenting emergency, we were limited to a pre/post design and thus, lacked a counterfactual control group. However, we found that confounding by potential temporal improvements in outcomes that may have occurred outside our intervention were unlikely, since baseline outcome measures among those enrolled during the second half of the rolling enrollment period, compared to those enrolled during the first half, were essentially unchanged. We are currently in the early part of a next-stage cluster randomized trial to take the evidence-base forward (Tucker et al., 2023). Second, collinearity between displacement status and delivery-type (in person vs virtual) limited our ability to evaluate the effectiveness of virtual delivery in the non-displaced population. The field situation affected mode of delivery and a more controlled design would allow for comparisons between virtual, in person and hybrid delivery modes. This has specific implications for scale-up of such interventions and the possibility to enhance reach when in-person provision is compromised by war. Third, mental health measures may be strengthened in future studies using validated and diagnostic scales. Finally, the small number of male participants limited our ability to generalize to a male population. Within this crisis, men were more likely to be engaged in active duty and thus less available for the intervention. For future studies, efforts could valuably encourage male participation. Avenues for expanding psychosocial support to men could be in the form of a dedicated provision, partner invitation, or outreach to venues where men may be available for recruitment, such as within a trained military chaplaincy. (Who is a military chaplain and what role does he play in war? [Internet] 2023).

In conclusion, this pre-post evaluation showed that participation in Hope Groups was associated with marked improvements in mental health, positive parenting, prevention of violence against children, and reported child wellbeing among displaced and non-displaced Ukrainians. The promising early effectiveness of *Hope Groups* is encouraging, given the extreme level of trauma that Ukrainian children and families are experiencing. Our research suggests that adaptations of the *Hope Groups* model might be promising /in conflict and potentially other crisis contexts.

## Funding

UK Research and Innovation (GCRF), LEGO Foundation, Oak Foundation, Moderna Charitable Foundation, private donor.

## Disclaimer

The findings in this report are those of the authors. They do not necessarily represent the official position of their respective organizations.

## CRediT authorship contribution statement

**Susan Hillis:** Writing – original draft, Visualization, Validation, Supervision, Methodology, Investigation, Funding acquisition, Data curation, Conceptualization. **Sydney Tucker:** Writing – review & editing, Writing – original draft, Visualization, Supervision, Methodology, Investigation, Funding acquisition, Formal analysis, Data curation. **Nicole Baldonado:** Writing – review & editing, Writing – original draft, Validation, Supervision, Project administration, Methodology, Investigation, Funding acquisition, Conceptualization. **Evgenia Taradaika:** Writing – review & editing, Supervision, Project administration, Methodology, Investigation, Conceptualization. **Lyudmyla Bryn:** Writing – review & editing, Validation, Supervision, Project administration, Methodology, Investigation, Conceptualization. **Svitlana Kharchenko:** Writing – review & editing, Supervision, Project administration, Methodology, Investigation, Conceptualization. **Tetiana Machabelii:** Writing – review & editing, Supervision, Project administration, Methodology, Funding acquisition, Conceptualization. **Roisin Taylor:** Writing – review & editing, Methodology, Investigation, Data curation. **Phil Green:** Writing – review & editing, Methodology, Investigation, Funding acquisition, Conceptualization. **Philip Goldman:** Writing – review & editing, Resources, Methodology, Investigation. **Isang Awah:** Writing – review & editing, Methodology, Investigation, Conceptualization. **Joshua Baldonado:** Writing – review & editing, Validation, Supervision, Project administration, Methodology, Investigation, Funding acquisition, Data curation, Conceptualization. **Praveen Gomez:** Writing – review & editing, Validation, Project administration, Methodology, Investigation, Data curation, Conceptualization. **Seth Flaxman:** Writing – review & editing, Visualization, Methodology, Formal analysis, Conceptualization. **Oliver Ratmann:** Writing – review & editing, Visualization, Supervision, Methodology, Investigation, Formal analysis. **Jamie M. Lachman:** Writing – review & editing, Resources, Methodology, Investigation. **Andres Villaveces:** Writing – review & editing, Validation, Methodology, Conceptualization. **Lorraine Sherr:** Writing – review & editing, Methodology, Investigation, Conceptualization. **Lucie Cluver:** Writing – review & editing, Validation, Methodology, Investigation, Funding acquisition, Conceptualization.

## Declaration of competing interest

The authors declare the following financial interests/personal relationships which may be considered as potential competing interests: Prof Lucie Cluver reports financial support was provided by UK Research and Innovation Global Challenges Research Fund. Prof Lucie Cluver reports financial support was provided by The Oak Foundation. Jamie Lachman reports financial support was provided by Parenting for Lifelong Health. Jamie Lachman reports financial support was provided by University of Oxford. Jamie Lachman reports financial support was provided by LEGO Foundation. Jamie Lachman reports financial support was provided by The Oak Foundation. Prof Lucie Cluver reports a relationship with Univeristy of Oxford that includes: funding grants. Jamie Lachman reports a relationship with Parenting for Lifelong Health that includes: employment and funding grants. If there are other authors, they declare that they have no known competing financial interests or personal relationships that could have appeared to influence the work reported in this paper.

## Appendix Supplementary materials

Image, application 1
