# Supplementary material for: The effectiveness of Hope Groups, a mental health, parenting support, and violence prevention program for families affected by the war in Ukraine: Findings from a pre-post study
Source: J Migr Health. 2024 Jul 23;10:100251. doi: 10.1016/j.jmh.2024.100251 (PMC11340612; doi:10.1016/j.jmh.2024.100251)
Supplement: Supplementary file 1 [file mmc1.docx]

Supplementary Material: **The Effectiveness of *Hope Groups,* a Mental Health, Parenting Support, and Violence Prevention Program for Families Affected by the War in Ukraine: Findings from a Pre-Post Study**

**Table of Contents**

Diagram: Hope Groups Conceptual Model………………………………………………………………………2

Supplementary Table S1. Survey Measures………………………………………………………………….….3

Supplementary Table S2. Adjusted Linear Regression Models .………………………………………………5

Supplementary Table S3. Assessing Effect Measure Modification in Hope Group Participant Outcome Improvements by Training of Facilitator (Lay-Trained or Mental Health Professional).…………………...…6

Supplementary Table S4. Sensitivity Analysis: Poisson Models….…………………………………………….7

Supplementary Table S5. Analysis of Time Trends: Comparing Baseline Measures Over Time …………..8

**Diagram. Hope Groups Conceptual Model**

**Caregiver Wellbeing**

**Acute & Protracted War Exposure**

**Displacement Stressors**

**Re-Entry Stressors**

**Hope Groups**

**Hope Groups Conceptual Model**. The *Hope Groups* conceptual model shows that the intervention aims to strengthen caregiver mental health, parenting skills, and child protection skills, all of which may be adversely affected by war exposures and displacement stressors. By strengthening caregiver mental talk, parenting and child protection skills, the conceptual model shows that key aims are to reduce violence against children, and to improve both children’s and caregivers’ wellbeing. Informed by Global Reference Group for Children in Crisis (https://www.spi.ox.ac.uk/the-global-reference-group-on-children-affected-by-covid-19) Annual Strategy Meeting, World Bank, August 29-29, 2023), and with inspiration from Miller KE, Koppenol-Gonzalez GV, Arnous M, Tossyeh F, Chen A, Nahas N, et al. Supporting Syrian families displaced by armed conflict: A pilot randomized controlled trial of the Caregiver Support Intervention. *Child Abuse & Neglect*. 2020;106:104512. doi:10.1016/j.chiabu.2020.104512.

**Children’s Wellbeing**

**Violence against Children**

**Caregiver Mental Health**

**Parenting Skills**

**Child Supervision and Protection Skills**

| **Supplementary Table S1. Survey Measures** | | |
| --- | --- | --- |
| Outcome | Specific Question | Adapted from |
| **Depression** | *How many days in the past week did you feel depressed, or like everything was an effort? [0-7]* | CES-D Depression Screening Tool |
| **Self Care Practices** | *How many days in the past week did you take actions to care for yourself, such as relaxation techniques, talking to someone you trust about your feelings, establishing a routine, or eating and sleeping well? [0-7]* | Created for Ukrainian context |
| **Hopefulness about Future** | *How many days in the past week did you feel hopeful about the future? [0-7]* | Center for Epidemiological Studies Depression |
| **Coping with Grief** | *How many days in the past week were you able to grieve your losses in a healthy way, such as by talking with someone you trust, finding ways to honor who or what you lost, or finding meaning in your new reality? [0-7]* | Created for Ukrainian context |
| **Physical Violence** | *How many days in the past week did you discipline your child physically, such as hitting with your hand, stick, or belt? [0-7]* | International Society for the Prevention of Child Abuse and Neglect Screening Tool – Physical Abuse Subscales |
| **Emotional Violence** | *How many days in the past week did you shout or scream at your child? [0-7]* | International Society for the Prevention of Child Abuse and Neglect Screening Tool – Emotional Abuse Subscales |
| **Child Despondency** | *How many days in the past week did your child seem unhappy, downhearted, or tearful? [0-7]* | Created for Ukrainian context |
| **Child Verbalizing Emotions** | *How many days in the past week was your child able to talk about their problems and share their worries? [0-7]* | Parent Child Communications Scale |
| **Monitoring Child** | *How many days in the past week did you know who your child spent time with, both online and in person? [0-7]* | Parent Protective Practices Scale |
| **Reinforcing Positive Behavior** | *How many days in the past week did you praise something your child did well? [0-7]* | Alabama Parenting Questionnaire - Positive Parenting and Parental Involvement subscales |
| **Supporting Child Development through Play** | *How many days in the past week did you play with your child(ren), or help them learn something new? [0-7]* | Alabama Parenting Questionnaire - Positive Parenting and Parental Involvement subscales |
| **Protecting Child** | *How many days in the past week did you do something to keep your child safe, such as make sure they don't spend time outside alone after dark, or take a ride/lift with someone alone? [0-7]* | Parent Protective Practices Scale |
| **Nonviolent Discipline** | *How many days in the past week did you try to speak calmly with your child when you were upset with them? [0-7]* | Parenting Young Children |

|  | **Supplementary Table S2: Adjusted Linear Regression Models* Comparing Mean Difference in Days** | | | | | | | |  |  |
| --- | --- | --- | --- | --- | --- | --- | --- | --- | --- | --- |
|  |  | Midline Estimate  (95% CI) | |  | Midline P-Value |  | Endline Estimate (95% CI) | |  | Endline P-Value |
|  | **Mental Health Outcomes among Full Sample (N=577)** |  |  | |  |  |  |  |  |  |
|  | Depression | -1.23 | -1.4, -1.06 | | < .001*** |  | -1.86 | -2.03, -1.69 |  | < .001*** |
|  | Self Care Practices | 1.23 | 1.05,1.4 |  | < .001*** |  | 2.15 | 1.97, 2.33 |  | < .001*** |
|  | Coping with Grief | 0.88 | 0.67,1.1 |  | < .001*** |  | 1.44 | 1.23, 1.66 |  | < .001*** |
|  | Hopefulness about Future | 1.12 | 0.94,1.29 |  | < .001*** |  | 2.09 | 1.91, 2.26 |  | < .001*** |
|  | **Parenting and Child Health Outcomes among Co-residing Parents and Caregivers (N=381)** |  |  | |  |  |  |  |  |  |
|  | Physical Violence | -0.13 | -0.24, -0.03 | | 0.01** |  | -0.25 | -0.36, -0.14 |  | < .001*** |
|  | Emotional Violence | -0.75 | -0.92, -0.58 | | < .001*** |  | -1.28 | -1.45, -1.11 |  | < .001*** |
|  | Supporting Child Development | 0.61 | 0.41,0.81 |  | < .001*** |  | 1.69 | 1.49, 1.89 |  | < .001*** |
|  | Monitoring Child | 0.81 | 0.6,1.01 |  | < .001*** |  | 1.72 | 1.51, 1.93 |  | < .001*** |
|  | Protecting Child | 0.62 | 0.41,0.84 |  | < .001*** |  | 1.43 | 1.21, 1.65 |  | < .001*** |
|  | Nonviolent Discipline | 0.54 | 0.29,0.78 |  | < .001*** |  | 1.1 | 0.83, 1.35 |  | < .001*** |
|  | Reinforcing Positive Behavior | 0.62 | 0.43,0.82 |  | < .001*** |  | 1.58 | 1.38, 1.77 |  | < .001*** |
|  | Child Verbalizing Emotions | 0.91 | 0.66,1.16 |  | < .001*** |  | 1.57 | 1.32, 1.82 |  | < .001*** |
|  | Child Despondency | -0.77 | (-0.94, -0.59) | | < .001*** |  | -1.2 | -1.39, -1.02 |  | < .001*** |

* Each model adjusted for age and sex and including random effects on participant and facilitator to account for clustering

| **Supplementary Table S3. Assessing Effect Measure Modification in Hope Group Participant Outcome Improvements by Training of Facilitator (Lay-Trained or Mental Health Professional)** | | | | | | | | | | | |
| --- | --- | --- | --- | --- | --- | --- | --- | --- | --- | --- | --- |
|  | Intervention Effect among Lay-Trained Facilitators | | | | | *Additional* Intervention Effect among Mental Health Professional Facilitators | | | | | |
|  | Midline Estimate (95% CI) | Midline P-vae | Endline Estimate (95% CI) | | Endline P-value | Midline Interaction Estimate (95% CI) | | Midline Interaction P-value | Endline Interaction Estimate (95% CI) | | Endline Interaction P-value |
| **Mental Health Outcomes among Full Sample (N=577)** |  |  |  |  |  |  |  |  |  |  |  |
| Depression | -1.19 (-1.51, -0.89) | .<.001** | -1.48 | (-1.79, -1.18) | .<.001** | -0.13 | (-0.50, .23) | 0.48 | -0.58 | (-0.95, -0.22) | 0.002** |
| Self Care Practices | 1.06 (0.74, 1.38) | <.001*** | 1.38 | (1.06, 1.71) | .<.001** | 0.33 | (-.06, 0.71) | 0.09 | 1.08 | (0.70, 1.47) | <.001*** |
| Coping with Grief | 0.76 (0.38, 1.14) | <.001*** | 0.92 | (0.53, 1.31) | .<.001** | 0.25 | (-0.21,0.71) | 0.29 | 0.78 | (0.32, 1.25) | <.001*** |
| Hopefulness about Future | 0.87 (0.56, 1.18) | <.001*** | 1.53 | (1.21, 1.85) | .<.001** | 0.45 | (0.07 - 0.82) | 0.02* | 0.83 | (0.45, 1.24) | <.001*** |
| **Parenting and Child Health Outcomes Outcomes among Co-residing Parents and Caregivers (N=381)** |  |  |  |  |  |  |  |  |  |  |  |
| Physical Violence | -0.05 (-0.24, 0.13) | 0.58 | -0.33 | (-0.51, -0.14) | .<.001** | -0.12 | (-0.35, 0.10) | 0.28 | 0.09 | (-0.14, 0.31) | 0.45 |
| Emotional Violence | -0.56 (-0.86, -0.26) | <.001*** | -1.01 | (-1.31, -0.71) | .<.001** | -0.56 | (-0.86, -0.26) | 0.10* | -0.43 | (-0.79, -0.07) | 0.02* |
| Supporting Child Development | 0.34 (-0.0, 0.69) | 0.05* | 1.17 | (0.83, 1.52) | .<.001** | 0.41 | (0.05, 0.11) | 0.05* | 0.72 | (0.30,1.13) | <.001*** |
| Monitoring Child | 0.46 (0.09,0.83) | 0.02* | 1.29 | (0.92, 1.65) | .<.001** | 0.48 | (0.04, 0.92) | 0.03* | 0.52 | (0.07, 0.96) | 0.02* |
| Protecting Child | 0.59 (0.21, 0.96) | 0.002** | 0.95 | (0.57, 1.33) | .<.001** | 0.12 | (-0.33, 0.57) | 0.59 | 0.68 | (0.22, 1.13) | 0.004** |
| Nonviolent Discipline | 0.53 (0.1,0.96) | 0.02* | 0.64 | (0.21, 1.07) | .<.001** | 0.07 | (-0.45, 0.59) | 0.78 | 0.66 | (0.14, 1.19) | 0.01* |
| Reinforcing Positive Behavior | 0.45 (0.11, 0.79) | <.001** | 1.03 | (0.69, 1.37) | .<.001** | 0.32 | (-0.09, 0.72) | 0.13 | 0.78 | (0.38, 1.19) | <.001*** |
| Child Verbalizing Emotions | 1.03 (0.59, 1.46) | .<.001** | 1.21 | (0.77, 1.65) | .<.001** | 0.09 | (-0.62, 0.44) | 0.73 | 0.51 | (-0.02, 1.04) | 0.06 |
| Child Despondency | -0.54 (-0.86, -0.23) | .<.001** | -1.01 | (-1.32, -0.69) | .<.001** | -0.38 | (-0.76, 0.0) | 0.05* | -0.37 | (-0.75, 0.01) | 0.06 |

*Indicates level of significance. *Models were not adjusted for covariates, in order to yield both the estimated change in outcomes within the referent group (lay-trained) and the additional change within the groups led by professionally trained facilitators.*

| **Supplementary Table S4. Poisson Regression Models Indicating Incidence Rate Ratios (IRR) at Midline and Endline of Participation in Hope Groups** | | | | | | | | | |
| --- | --- | --- | --- | --- | --- | --- | --- | --- | --- |
|  | Midline IRR (95% CI) | |  | Midline P-Value | | Endline IRR (95% CI) | |  | Endline P-Value |
| **Mental Health Outcomes** among Full Sample **(N=577)** |  |  |  |  |  |  |  |  |  |
| Depression | 0.62 | (0.57,0.66) |  | <.001*** |  | 0.43 | (0.4,0.47) |  | <.001*** |
| Self Care Practices | 1.42 | (1.34,1.51) |  | <.001*** |  | 1.72 | (1.63,1.82) |  | <.001*** |
| Coping with Grief | 1.39 | (1.3,1.48) |  | <.001*** |  | 1.62 | (1.52,1.72) |  | <.001*** |
| Hopefulness about Future | 1.35 | (1.28,1.42) |  | <.001*** |  | 1.61 | (1.53,1.70) |  | <.001*** |
| **Parenting and Child Health Outcomes** Outcomes among Co-residing Parents and Caregivers **(N=381)** |  |  |  |  |  |  |  |  |  |
| Physical Violence | 0.67 | (0.52,0.86) |  | .002** |  | 0.32 | (0.23,0.45) |  | <.001*** |
| Emotional Violence | 0.66 | (0.6,0.73) |  | <.001*** |  | 0.42 | (0.37,0.47) |  | <.001*** |
| Playful Parenting | 1.14 | (1.07,1.21) |  | <.001*** |  | 1.39 | (1.31,1.48) |  | <.001*** |
| Monitoring Child | 1.16 | (1.09,1.23) |  | <.001*** |  | 1.33 | (1.26,1.41) |  | <.001*** |
| Protecting Child | 1.11 | (1.05,1.18) |  | <.001*** |  | 1.26 | (1.19,1.33) |  | <.001*** |
| Nonviolent Discipline | 1.12 | (1.05,1.2) |  | <.001*** |  | 1.25 | (1.18,1.33) |  | <.001*** |
| Reinforcing Positive Behavior | 1.13 | (1.06,1.2) |  | <.001*** |  | 1.33 | (1.25,1.4) |  | <.001*** |
| Child Verbalizing Emotions | 1.27 | (1.18,1.35) |  | <.001*** |  | 1.43 | (1.34,1.53) |  | <.001*** |
| Child Despondency | 0.67 | (0.6,0.74) |  | <.001*** |  | 0.48 | (0.43,0.53) |  | <.001*** |

*Indicates level of significance.

*Models were not adjusted for covariates, as models with covariates would not converge. Adjusted models shown in Table S3 demonstrate results remain consistent after adjustment.*

| **Supplementary Table S5. Assessment of Time Trends: Comparing Baseline Measures During First Half and Second Half of ‘Hope Group’ Rolling Study Enrollment** | | | |
| --- | --- | --- | --- |
|  | **Baseline Measures from November 2022 - March 2023** | **Baseline Measures from March 2023 - July 2023** | **P-value** |
|  | **(N=340)** | **(N=362)** |  |
| **Depression** |  |  |  |
| Mean (SD) | 3.31 (2.12) | 3.22 (2.18) | 0.59 |
| Median [Min, Max] | 3.00 [0, 7.00] | 3.00 [0, 7.00] |  |
| **Physical Violence** |  |  |  |
| Mean (SD) | 0.362 (0.946) | 0.407 (1.09) | 0.63 |
| Median [Min, Max] | 0 [0, 7.00] | 0 [0, 7.00] |  |
| **Emotional Violence** |  |  |  |
| Mean (SD) | 2.29 (1.99) | 2.19 (1.90) | 0.62 |
| Median [Min, Max] | 2.00 [0, 7.00] | 2.00 [0, 7.00] |  |
| **Child Despondency** |  |  |  |
| Mean (SD) | 2.34 (1.82) | 2.30 (1.81) | 0.81 |
| Median [Min, Max] | 2.00 [0, 7.00] | 2.00 [0, 7.00] |  |
| **Self Care Practices** |  |  |  |
| Mean (SD) | 3.06 (2.15) | 2.83 (1.99) | 0.14 |
| Median [Min, Max] | 3.00 [0, 7.00] | 3.00 [0, 7.00] |  |
| **Coping with Grief** |  |  |  |
| Mean (SD) | 2.29 (1.99) | 2.48 (2.16) | 0.22 |
| Median [Min, Max] | 2.00 [0, 7.00] | 2.00 [0, 7.00] |  |
| **Hopefulness about Future** |  |  |  |
| Mean (SD) | 3.26 (2.39) | 3.58 (2.47) | 0.08 |
| Median [Min, Max] | 3.00 [0, 7.00] | 3.00 [0, 7.00] |  |
| **Supporting Child Development** |  |  |  |
| Mean (SD) | 3.96 (2.25) | 4.36 (2.25) | 0.06 |
| Median [Min, Max] | 4.00 [0, 7.00] | 4.00 [0, 7.00] |  |
| **Monitoring Child** |  |  |  |
| Mean (SD) | 4.59 (2.48) | 4.78 (2.35) | 0.4 |
| Median [Min, Max] | 5.00 [0, 7.00] | 5.00 [0, 7.00] |  |
| **Protecting Child** |  |  |  |
| Mean (SD) | 4.91 (2.51) | 5.08 (2.32) | 0.43 |
| Median [Min, Max] | 7.00 [0, 7.00] | 7.00 [0, 7.00] |  |
| **Nonviolent Discipline** |  |  |  |
| Mean (SD) | 4.01 (2.13) | 4.23 (2.27) | 0.28 |
| Median [Min, Max] | 4.00 [0, 7.00] | 4.00 [0, 7.00] |  |
| **Reinforcing Positive Behavior** |  |  |  |
| Mean (SD) | 4.48 (2.06) | 4.51 (2.24) | 0.86 |
| Median [Min, Max] | 4.00 [0, 7.00] | 5.00 [0, 7.00] |  |
| **Child Verbalizing Emotions** |  |  |  |
| Mean (SD) | 3.56 (2.35) | 3.48 (2.50) | 0.7 |
| Median [Min, Max] | 3.00 [0, 7.00] | 3.00 [0, 7.00] |  |
